# A Complete Set of Nascent Transcription Rates for Yeast Genes

**DOI:** 10.1371/journal.pone.0015442

**Published:** 2010-11-16

**Authors:** Vicent Pelechano, Sebastián Chávez, José E. Pérez-Ortín

**Affiliations:** 1 Departamento de Bioquímica y Biología Molecular, Facultad de Ciencias Biológicas, Universitat de València, Burjassot, Spain; 2 Departamento de Genética, Facultad de Biología, Universidad de Sevilla, Sevilla, Spain; University of Medicine and Dentistry of New Jersey, United States of America

## Abstract

The amount of mRNA in a cell is the result of two opposite reactions: transcription and mRNA degradation. These reactions are governed by kinetics laws, and the most regulated step for many genes is the transcription rate. The transcription rate, which is assumed to be exercised mainly at the RNA polymerase recruitment level, can be calculated using the RNA polymerase densities determined either by run-on or immunoprecipitation using specific antibodies. The yeast *Saccharomyces cerevisiae* is the ideal model organism to generate a complete set of nascent transcription rates that will prove useful for many gene regulation studies. By combining genomic data from both the GRO (Genomic Run-on) and the RNA pol ChIP-on-chip methods we generated a new, more accurate nascent transcription rate dataset. By comparing this dataset with the indirect ones obtained from the mRNA stabilities and mRNA amount datasets, we are able to obtain biological information about posttranscriptional regulation processes and a genomic snapshot of the location of the active transcriptional machinery. We have obtained nascent transcription rates for 4,670 yeast genes. The median RNA polymerase II density in the genes is 0.078 molecules/kb, which corresponds to an average of 0.096 molecules/gene. Most genes have transcription rates of between 2 and 30 mRNAs/hour and less than 1% of yeast genes have >1 RNA polymerase molecule/gene. Histone and ribosomal protein genes are the highest transcribed groups of genes and other than these exceptions the transcription of genes is an infrequent phenomenon in a yeast cell.

## Introduction

Gene transcription in eukaryotes is a complex process that starts with the recruitment of an RNA polymerase (RNA pol) complex to the gene promoter and is followed by a set of successive steps, such as initiation, elongation, splicing, termination, mRNA export, and degradation. Although it is well known that all of these steps are subject to strict regulation [1] the main objective of most regulatory studies is just the determination of the mRNA amount (RA) without being able to discriminate which steps are actually being regulated. RA can be easily measured by northern and RT-PCR techniques. Moreover, with the emergence of genomic techniques thousands of mRNAs can be simultaneously evaluated at the same time by DNA chip techniques [2] or by other more quantitative methods [3], [4]. However, the RA is the result of two opposite reactions, transcription and mRNA degradation, that can be characterized by chemical kinetic rates (the transcription rate, or TR, and the degradation rate) [5]. The main regulatory step for the gene expression of many genes is the control of their TR, which is assumed to be exercised mainly at the RNA pol recruitment level. Thus, variation in the mRNA level is usually attributed to changes in RNA pol recruitment to the promoter, and it is used to construct models in which transcription factors, nucleosome and histone modifications, among others, are the main players in the gene regulation game. However, as the regulation at the mRNA stability level is increasingly recognized to be important in gene regulation [5]–[8], the mRNA measurement can no longer be used as a direct estimation of gene transcription. Therefore, the existence of a complete set of TRs for a given organism would be of enormous interest for many researchers. TR can be mathematically calculated from RA and mRNA stability assuming steady-state conditions for gene expression [5]. In fact, the use of this kind of TR dataset has become very popular for yeast since Holstege *et al.* [9] provided a set of TR data as a supplementary material of that paper. Those data represent, however, the indirect calculation of the rate of appearance of mature mRNAs in the cytoplasm, taking into account all possible posttranscriptional processes of the mRNA, and do not represent the actual synthesis of new mRNAs by RNA pol in the genes (i.e. nascent TR).

We [10] and others [11], [12] have developed genomic variants of the well-known run-on technique [13] to evaluate the nascent TR for most genes. In this technique (GRO, Genomic Run-on), elongating RNA pol molecules, that conserve the RNA, are forced to incorporate radioactive UTP for a short length. The macroarray analysis of the *in vivo* labeled RNA measures the density of RNA polymerases in the analyzed genes that can be converted into TRs for all the yeast genes [10]. Like all experimental measurements, GRO is affected by an unavoidable precision error (random) and, potentially, by technical or biological biases (not random). Therefore, in order to improve the TR data obtained from GRO experiments, we have reduced the random error by increasing the number of biological repeats. Moreover, to decrease technical specific biases, we have used data from chromatin immunoprecipitation assay (ChIP) of RNA pol II inside the genes with specific antibodies (RNA Pol-ChIP-on-chip, RPCC) to detect and correct technical biases specifically associated to the GRO data and not present in the RPCC data. We also have incorporated the new estimations available for RA and stability and taken into account the dilution effect on the mRNA concentration due to the continuous increase of the total cellular volume during the exponential growth. All this has allowed us to obtain a reliable complete dataset for all the yeast gene nascent transcription rates for the first time in an eukaryote. We analyze this dataset and discover that histone genes are the most highly transcribed whereas most of yeast genes are scarcely transcribed. In fact, only 14% of them have an active RNA pol II molecules at a given moment and only a small proportion of RNA pol II molecules (6%) are actually productively transcribing the about 5900 ORF-containing *S. cerevisiae* genes.

## Results and Discussion

### Estimation of transcription rates using improved and corrected GRO values

When we first described the GRO method [10], we calculated the TRs for 5886 yeast genes, these being the vast majority of the RNA pol II genes. In that case, the values obtained for the TR from three individual samples of those cells exponentially growing in YPD medium in arbitrary units were converted into real ones (molecules/min) using the single published TR value of the *HIS3* gene [14]. That was the very first description of “nascent” TR dataset for an organism. The data we currently hold have been improved in five ways.

First, the current data are the averaged calculations from 24 individual samples exponentially growing in YPD to DO_600_ of 0.45. Second, they have been corrected for a probe-length artifact occurring in GRO data (supplementary Figure S1). This artifact was discovered when comparing individual data between GRO and RPCC. Both showed a bias with regard to gene length [15]. The TR was seen to decrease slightly with the gene length in the RPCC, while a stronger tendency was observed in the GRO. We found that this strong dependence in length of GRO is due to a bias that increases the labeling towards the 3′ end of the transcript and we hypothesize that it is due to the downstream movement of RNA polymerases (see supplementary text S1 and supplemental Figure S1 for a detailed description). Consequently we have used the RPCC data, which do not present this bias, to correct it.

Third, we used an updated total amount of mRNA molecules per yeast cell that has been corrected from that previously used (15000 molecules from ref. [16]) to more recent and precise calculations obtained from massive parallel sequencing and competitive PCR transcriptome measurements: 26000 molecules from refs. [3], [4]. Fourth, we used a more robust reference to convert TR into real units. We reasoned that because the *HIS3* TR was calculated mathematically by dividing the RA by mRNA stability [14], we could do a similar calculation for many more yeast genes if we used published data [3], [4], [17]. Therefore, we used the 2000 genes with the highest (and, therefore, the most confident) indirect TR value (corresponding, thus, to an “increase of mature mRNA TR” dataset, see below) to plot them against the averaged and corrected nascent TR dataset in arbitrary units in order to convert them into real units (molecules/min).

Finally, we have taken into account the effect of the dilution of the mRNA concentration due to the cell division. The dilution has a negative effect on the mRNA concentration and it is usually overlooked on the kinetic analysis of the transcription. However, as the volume of the cellular population is continuously increasing this effect, as well as the mRNA degradation itself, has to be compensated by the TR. In the standard mRNA stability calculations this factor is not taken into account because the cells' growth is inhibited by the treatment used to stop the transcription [6], [17]. For computing an indirect transcription rate it is necessary to use the mRNA amount (RA) and the rate of decrease of mRNA concentration. This decrease rate is due to both the mRNA degradation and to the dilution effect. The decrease in the RA due to the cellular dilution is directly proportional to the RA of each individual gene and inversely proportional to the generation time [RA*(ln2/generation time)]. To incorporate this factor we modified the indirect TR data (that were computed with non-growing cells) by adding this factor. Afterwards, we used the modified indirect TR dataset to convert the nascent TR data into real units. In this way, we have obtained an improved, highly confident, TR dataset for 4670 yeast genes in standard growing conditions (Table S1).

### Advantages of the nascent TR dataset

**Figure 1:**
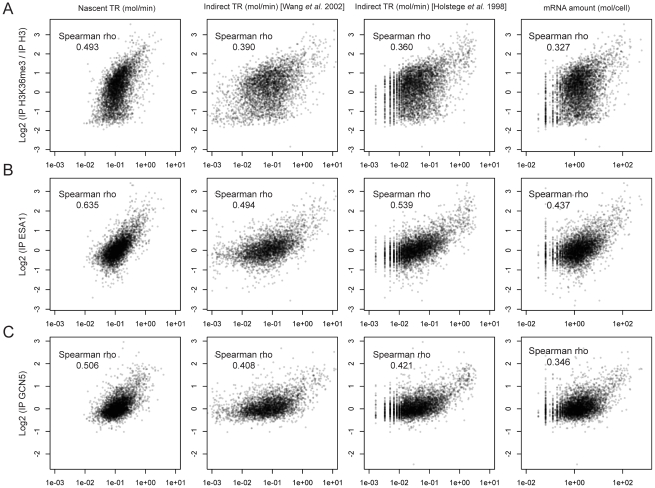
Comparison of the direct and indirect TR datasets with modifications related to elongation activity. Comparison of the new nascent TR dataset (this study) with a newly computed indirect TR datasets using the mRNA amount and the mRNA stability [17] that takes into account the effect of the dilution (see discussion), the indirect TR dataset from [9], and mRNA amount with different chromatin related parameters associated with active elongation as H3 trimethylation (H3K36me3, A) or presence of elongation related transcription factors the histone acetyltranferases Esa1p [27] (B) or Gcn5p (C) [25]. To avoid any bias depending on the wideness of the datasets Spearman rank correlation is used. Note that the correlations are always higher for the nascent TR dataset than for the indirect datasets, and that the indirect TR datasets show a better correlation than the mRNA amount data set.

**Figure 2:**
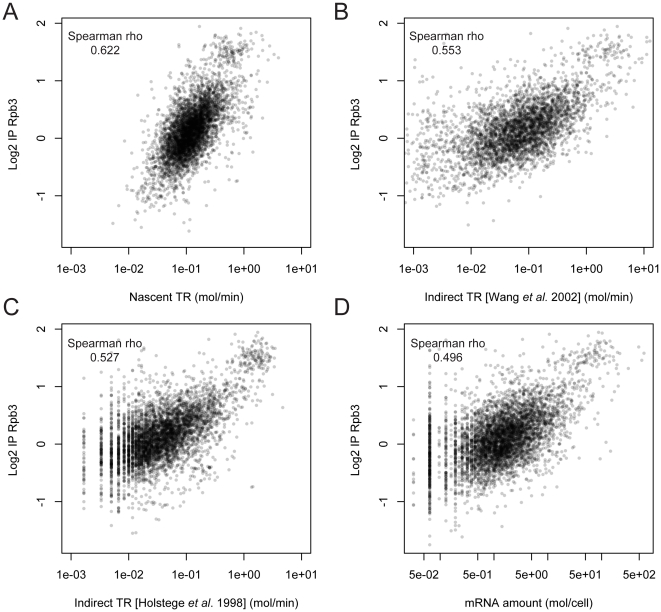
Comparison of the direct and indirect TR datasets with presence of RNA pol II. Comparison of the new direct TR dataset (this study) and indirect TR datasets computed using the mRNA amount and the mRNA stability [9], [17] as in Fig. 1 with RNA pol II data (average IP along the coding region of Rpb3-TAP) [18]. To avoid any bias depending on the wideness of the datasets Spearman rank correlation is used. Note that the correlations are always higher for the nascent TR dataset (A) than for the indirect datasets (B, C), and that the indirect TR datasets show a better correlation than the mRNA amount data set (D).

Until now many researchers have used an alternative TR dataset based on indirect calculations. In 1998 Holstege *et al.* [9] used an RA data set divided by “apparent half-life” data to obtain an apparent “transcriptional frequency”, offered in the supplementary information at the authors' web site. This “apparent half-life” was obtained from the slope of decay curves that had been adjusted with just two experimental time points. Despite the uncertainty associated with indirect calculations and the use of poor half-life data, those data are still the most widely used in the literature as a source of yeast TR, probably because they positively correlate with the expected enrichment of the transcribed genes in some histone modifications or in active gene-associated proteins [18]–[20]. The advantage of this indirect approach to calculate TR is that it requires no experimental method and that it does not need to assume a constant RNA pol speed on genes to convert RNA pol II densities into TRs. However, it does present other possible drawbacks: 1) it relies on the hypothetical assumption of steady-state conditions, 2) the mathematical calculation of the data could increase the experimental error and, 3) the need to measure the mRNA disappearance during a prolonged time provokes a stressful situation that affects genes expression. Moreover, as the mRNA stabilities are computed in cells that are not actually growing, the dilution rate of the cellular content due to the cell growth, that is compensated by increasing the actual TR, is not taken into account. This indirect TR set, therefore, only accounts for the part of the TR that is devoted to compensate mRNA degradation. We have discarded the first potential drawback verifying the steady-state hypothesis during exponential growth in glucose [21]. However, the other error sources are unavoidable. As the indirect TR data are based on the measurement of mature mRNA disappearance, and thus it takes into account also all the posttranscriptional processes as well as the RNA degradation, the direct nascent TR data could be a better indicator of the effects of RNA pol II transcription on the *in vivo* gene chromatin structure features, such as histone modifications, transcription factor binding or nucleosome positioning [18]–[20], [22]–[27]. We have verified (see Figure 1) that our nascent TR dataset is better correlated with chromatin marks [25] directly related with transcription elongation, such as H3K36me3 that is deposited during RNA polymerase elongation [26] or with the presence of histone acetyltransferases related to elongation such as Gcn5p or Esa1p [27]. And it is, at least, as equally well correlated to chromatin modifications associated with active promoters [25] as the indirect TR data (see supplemental Figure S2). For these comparisons we have also used the indirect TR provided in [9] as well as a new set of indirect TR that we computed using more accurate and recent mRNA stability [17] and RA [3], [4] (see details in Methods section). Moreover, we have repeated the same analyses using just the RA instead of TR. It can be seen that for transcription elongation markers indirect TR is slightly better correlated than RA and that the improvement caused by the use of nascent TR is much higher (Figure 1). With regard to non-elongating marks the results for all the datasets are much more similar (Figure S2). These comparisons demonstrate that the actual presence of active RNA pol II on genes, quantified by the nascent TR dataset, is the best predictor of elongating-related chromatin features. This fact has been also confirmed by the direct comparison between the different TR datasets with RNA pol II occupancy (Figure 2): nascent TR dataset is also better correlated with the actual presence of RNA pol II, as determined by chip-on-chip analysis of the Rpb3 subunit [18].

It should be noted that all the nascent TR evaluations based on active RNA pol II density measure could differ from the increase of mature mRNA (Figure S3) owing to the fact that either some of the elongating polymerase molecules never reach the end of the gene or some of the nuclear pre-mRNAs are degraded [28]. This can differentially affect yeast genes, thus introducing a source of discrepancy with regard to those indirect TR calculations. In fact, it would be possible to use this discrepancy to study the different way in which the genes are expressed. To prove that our direct nascent TR data measures directly the transcription process, and thus differs from the indirect estimation of increase of mature mRNA concentration we compared both TR datasets with regard to the transcription features in which discrepancies are expected. First, our nascent transcription rates have not strand-specificity, comprising the transcription for both the sense and any hypothetical antisense or cryptic transcription. As most of the antisense and cryptic transcripts are degraded in the nucleus it is expected that the genes with known antisense transcription (CUTs or SUTs) [29] present a higher nascent TR over their indirect TR. In fact the relationship between nascent and indirect transcription is significant higher for those genes (Figure S4). This confirms that the nascent TR is able to detect the transcription process independently of the stability of the produced transcripts. Allowing thus to monitor directly the transcription process itself without taking into account any posttranscriptional process.

Second, Venters and Pugh [24] have recently proposed that some yeast genes (1070 genes called “Group 2”) might be regulated at the level of elongation because they have enriched levels of RNA pol II molecules on their 5′ end transcribed region. We analyzed the behavior of the 652 genes of this paused RNA pol II class present in our nascent TR dataset (Table S1 and Figure S5) and, as expected and according to their proposal, we found a significant excess of nascent TR over the indirect TR only in these genes (comparing the log_2_ lowess corrected values (see Methods section), t-test with sig. 4.71·10^−4^). In opposition, the 561 genes of group 1 (characterized by accumulation of non-elongating polymerases in the promoter region) showed less nascent TR over the indirect TR (sig. 8.24·10^−7^). We do not know the reason of this but it could be explained if these genes have less cryptic transcription or a more efficient maturation and export process. Finally, there is no significant difference (sig. 0.93) between nascent and indirect TR for the 560 genes of group 3 that has an even or enriched to the 3′ distribution of polymerases.

### Analyses of the nascent TR dataset

**Figure 3:**
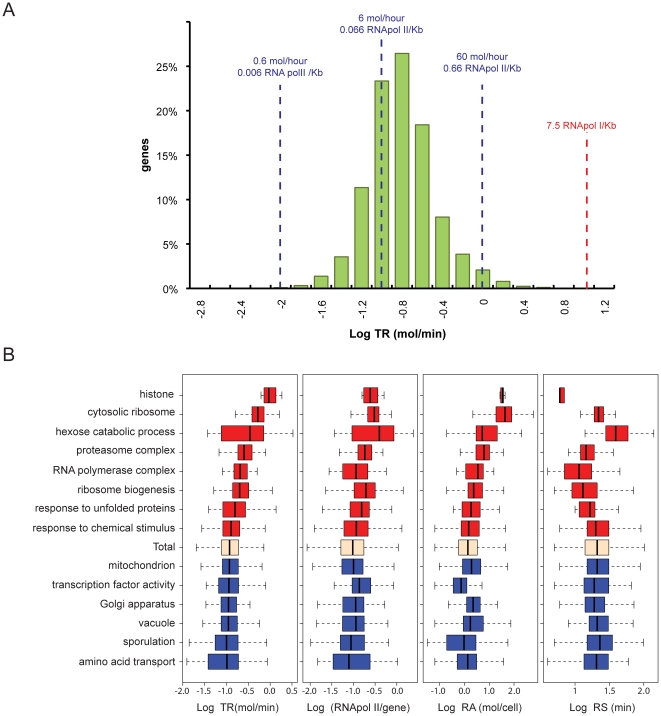
Nascent TR data set. A) Histogram of the yeast nascent productive TR data set in real units (molecules per hour, mol/hour) and RNA pol II densities (RNA pol II molecules per kb, mol/kb) for all the genes. The RNA pol I density [32] is shown as a reference with a dashed red line. The natural values of RNA pol II density and TR corresponding to −2, −1 and 0 units in log scale are shown as dashed blue lines. B) Comparison of nascent TR, RNA pol II molecules per gene, RNA amount (RA, using RNA-Seq data corrected with GATC-PCR, see Methods) and mRNA stability from ref. [17] for the different selected groups of genes (GO categories). The box plots represent the median and quartiles of the data. The whiskers show the maximum and minimum of the data set (excluding the outliers which lie beyond 1.5 times the inter quartile range).

The distribution of the TR values (Figure 3) is similar to a log-normal as shown in most cases by the expression datasets, being the median TR about 0.12 mRNA molecules/min (equivalent to 7 mRNAs/hour). Furthermore, 90% of the genes have TRs between 2.33 and 29.7 mRNAs/hour and, if we assume that the 4670 genes for which we have data are representative of the 5796 non-dubious ORFs, the total transcription for RNA pol II in a yeast cell growing in standard conditions is about 60200 mRNAs/h. By assuming the known datum that RNA polymerase molecules transcribe at 25 nt/s [30], then the median RNA pol II density inside the genes is 0.078 molecules/kb.

Gene functional groups tend to have similar TRs (Fig. 3B first graph), which reflects that coordinated transcription is one of the main steps in coordinating gene expression. However, some of GO categories, e.g. the hexose catabolism genes, present a wider range of TRs. This can reflect that the coordination of the biological function is less demanding at the transcriptional level, that there is strong control in a later expression step or, simply, that the GO category is less strictly defined. For most groups, the average position within the whole population in RA and TR is similar (Fig. 3B, compare first and third graphs). An exception to this is the group of histone genes. Their relatively high RA value is the result of the highest average TR level that is compensated by quite unstable mRNAs (Fig. 3B fourth graph). This group follows an expression strategy with very high TR and very low mRNA stability [7] that allows for fast changes in RA [5]. Histone genes are, thus, among the highest transcribed genes with a median TR of almost 56 mRNAs/hour. Moreover, because histone genes are only transcribed during the S phase, which represents only 27% of the cell cycle in exponentially growing yeast [31], the TR for actual active histone genes on an asynchronic cell population will be 206 mRNAs/hour that corresponds to a RNA pol II density of 2.29 molecules/kb. This is still 3 times lower than the RNA pol I density on rDNA active genes [32] and 4 times lower than the theoretical maximal density calculated just by using the physical size of the elongating RNA pol II and its maximal rate of initiation [33]. On the other hand, it can be seen also in Fig. 3 that sporulation is among the groups with the lowest TRs, coinciding with a very low RNA pol II density, as determined by Venters and Pugh [24]. Other group with very low median TR is amino acid transport (Figure 3B and Supplementary Figure S6). All these results are logical for cells exponentially growing in complete YPD medium.

**Figure 4:**
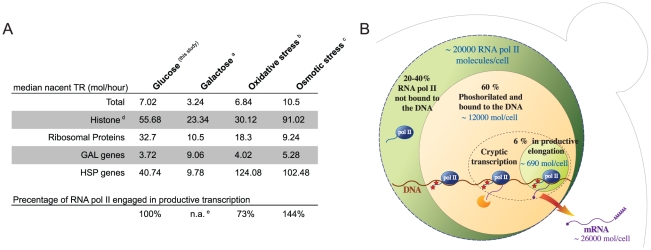
A snapshot of the yeast transcriptome. A) Selected data from the yeast nascent TR dataset. It is shown the median values for the TR for some selected groups of genes in exponential growth in YPD (glucose, this study) as well as in different conditions using previously published GRO datasets normalized with the newly computed nascent data: ^a^ cells growing exponentially in YPGal, (galactose: time 5 from [10]); **^b^** 16 min after the oxidative stress due to the addition of 0.1 mM t-BOOH [36] ^c^ 10 min in 0.4 M NaCl [37]. It is also shown the percentage of RNA pol II engaged in productive transcription (total transcription activity) relative to the exponential growth in glucose. ^e^ In the case of yeast cells growing in galactose this percentage is not significant because it is not possible to assume that the total amount of RNA pol II has not changed after several cell cycles using galactose as carbon source. On the other hand, we assume that the total amount of RNA pol II has not change during the short stresses. B) Schema of the yeast nucleus showing the computed distribution of transcriptional machinery during the exponential growing in rich medium. The size of the respective circles reflects the amount of RNA pol II molecules in each category.

Our results differ, in some aspects, from a previous study based in “mature mRNA appearance” by Struhl [33]. In that study the most transcribed genes in exponential growth in glucose were the ribosomal protein (RP) genes. However, in our exponential growth YPD nascent TR dataset RP genes show a median TR of 32.7 mRNAs/hour (Figures 3 and 4), six times lower than the histone genes. In fact, summing up all RP genes their transcription represents 8.5% of total RNA pol II transcription, which is much lower than previous estimations (50%) based on indirect calculations [34]. This is, in part, due to the fact that they exceed an amount of non-active RNA polymerases that reduces their effective TR (see ref. [35] for discussion). It was also discussed that *GAL* genes and stress–responsive genes have the highest TRs, obviously in growth conditions other than the exponential growth in YPD [33]. However, using our previously published data, we have computed the TR level of *GAL* genes in exponential growth in galactose [10] and of stress-responsive genes after oxidative [36] or osmotic stress [37] and we have found that *GAL* genes in YPGal medium have a median TR of about 10.5 molecules/hr and that stress-responsive genes after stress reach a median TR of 124 molecules/hr, less than the one of the histone genes in exponential growth in YPD (Figure 4A and Supplementary Table S2 ).

### Respective contributions of TR for compensating mRNA degradations rates and dilution rate

If we assume a steady-state situation for the exponential growth in YPD (demonstrated in [21]) the productive transcription rate should compensate the disappearance of mRNA occurring by mRNA degradation and by the dilution caused by continuous growth. Therefore, knowing the growth rate, it is possible to estimate the part of the TR devoted to compensate the mRNA degradation and the part devoted to compensate the dilution. In our data, obtained from exponentially growing cells in YPD with a doubling time of 113 minutes, although the dilution effect on the decrease of the mRNA concentration is much lesser than the mRNA decrease due to the mRNA degradation (only 8% on average), its contribution is not negligible and not identical for every gene. In fact, for some genes the dilution is the main factor for the mRNA disappearance and most of the transcriptional activity is devoted to compensate it (Supplementary Table S3). As the decrease due to the dilution is directly proportional to the mRNA amount for every gene this effect is more important for “housekeeping” genes that maintain a high RA and are relatively stable. The GO categories related with translation and biosynthetic processes are enriched in the group of genes in which TR devoted to compensate the dilution effect is more important (Supplementary Fig. S7). On the other hand, in the mRNAs expressed at low level and with low stability most of its transcription is devoted to compensate the mRNA degradation, and the effect of the dilution is almost negligible. This group is enriched in GO categories related with replication, transcription and mitosis (see Supplementary Figure S7).

### Transcription and chromatin

It is known that nucleosome stability and distribution are affected during transcription elongation [38], and it has been demonstrated that active genes could be partially depleted of H2A–H2B dimers and H3–H4 tetramers [39]. In fact, the active copies of rDNA gene are totally depleted of nucleosomes because of the steric hindrance caused by high density of RNA pol I molecules [40]. Our RNA pol II density calculations (see Fig. 3) predict that even in the case of the highest transcribed RNA pol II genes, theoretically, nucleosomes have the time and room required to re-form, at least, partially. That is, a RNA pol II complex will transcribe in histone genes during their maximum transcription (206 mRNAs/hour) every 17.5 seconds through the region occupied by a nucleosome. With a RNA pol II speed of 25 bp/s it takes around 6 seconds to pass through 147 bp. Thus the nucleosomes could be associated with the DNA for 65% of the time. This will cause a nucleosome density more than three times higher than that shown for fully activated *GAL* genes [39]. In that case, however, the authors demonstrated that there is a significant reduction in histone-DNA contact when the genes are activated [39], and this has been genomically extended to other active genes [41]. Therefore, it can been concluded that the effect of RNA pol II passage on chromatin extends for longer periods than the actual time required to read the nucleosome-wrapped DNA sequence [39], which confirms previous suggestions that, in highly transcribed RNA pol II genes, nucleosome organization and positioning are extensively altered [42] with the help of chromatin remodeling complexes [38], but without an important contribution of RNA polymerase steric hindrance. Because our results show that RNA pol II molecules are not so closely spaced, even in the highest transcribed genes, to sterically prevent DNA-octamer re-association behind the transcribing first RNA pol II molecule, the model that better fits the observations is that the next RNA pol II molecule could approach a partially remodeled nucleosome and displace it easily because of its lowered stability [38].

A possible error involved in the RNA pol II density calculated from the TR data is that the average speed for RNA pol II molecules is, perhaps, higher than 20–30 pb/s [43]. In fact, it has been recently shown that in human genes the average speed for many genes is about 63 pb/s [44]. Although, it has to be considered that this data was calculated at 37°C instead of the 28–30°C commonly used for yeast experiments. If the RNA pol II speed were higher the calculated densities for RNA pol II molecules would be even lower. Alternatively, if the number of mRNA molecules per cell were higher than 26000 as has also been suggested [43] the densities of the RNA pol II molecules would be higher. Therefore, we keep our calculations as they are in Table S1.

### A snapshot of the yeast cell transcription

It was said that transcription occurs multiple times in most yeast genes within a single cell cycle [33]. This opinion sees the glass half full. However, although our current results confirm this view, we see the glass half empty because most genes are productively transcribed just once or a few times per cell cycle and that most of the transcriptional machinery is not active at the same time. In fact we can say that productive transcription on a yeast gene is a rare phenomenon in an actively growing cell. In any case, it is necessary to stress that the conversion of the initial nascent TR values in arbitrary units into absolute TR is based in the comparison with the absolute values from the indirect dataset (see Methods section) that normalizes the total amount of transcription events only to those that finally produce a mature mRNA in the cytoplasm. This means that only the “productive” events of transcription are considered. Part of the transcribing RNA pol II molecules that are detected in GRO never will produce an mRNA because of getting arrested during transcription or because the mRNA will not be exported to the cytoplasm. With the current technology it is not possible to measure the fraction of “unproductive events” but if we take as indicator of the cryptic transcription the relative abundance of transcripts outside the coding regions, it does not seem likely that it represents a high percentage of the total RA [45]. However, it has to be taken into account that all these genome wide studies measure only the mature mRNA that has not been degraded and can be detected by microarray or RNAseq technology. To discard this we have computed the total signal due to CUTs (cryptic unstable transcripts) in a *rrp6* mutant from the experiment described in [29]. We found that cryptic transcripts in *rrp6* only represents around the 5% of the total RA signal. Thus, it can be assumed that the fraction of cryptic transcription should not be very high.

According to productive events the median elongating RNA pol II density of 0.078 molecules/kb corresponds to an average of 0.096 molecules/gene. The statistical distribution shows that less that 1% of yeast genes have >1 molecule of elongating RNA pol II/gene. Therefore, we calculate that about 690 RNA pol II molecules are actively transcribing in a snapshot of an average cell in exponential growth in YPD medium. This figure can be increased if we consider that part of the transcription events on real genes will not result in mature transcripts in the cytoplasm. For instance, we have preliminary evidence (Montón and Pérez-Ortín, unpublished) that almost half of the RNA pol II molecules that initiate elongation do not reach the end of the gene *YLR454w* when using the experimental protocol described by Mason and Struhl [28]. This fact matches the proposed model of “transcription factories” [46] which imply that, at least for short genes, two RNA pol II molecules are not simultaneously reading a gene. It has been shown by single molecule analysis that a yeast gene has a median distribution of 2 RNA pol II molecules on it [43]. The gene analyzed (*MDN1*) is, however, 14 kb long. Therefore, it is not contradictory with our conclusion. On the other hand, because our data are population averages it is very likely that some individual cells have higher densities in some genes.

According to the data discussed by Struhl [33] there are about 20000 RNA pol II molecules per cell (30000 according [47]) and 60% of those are hyperphosphorylated. On the other hand, the chromatin-associated RNA pol II molecules have been calculated to be between 60–80% using FRAP (fluorescence recovery after photobleaching) [48]. Therefore, at least 12000 RNA pol II molecules would be hyperphosphorylated in Ser2 or/and Ser5 of the carboxy terminal domain (CTD) and associated to genes but only 690–1380 (6–12% of them, depending on the proportion of mRNA nuclear degradation) would be actually transcribing the ORF-containing genes. This result coincides with the calculations made by Struhl [33] who estimated that 90% of the RNA pol II initiation events represent “transcriptional noise”. The rest of the hyperphosphorylated RNA pol II molecules would be either backtracked onto ORF-containing genes (see below) or transcribing onto other genome locations from either cryptic promoters which transcripts are not stabilized in a *rrp6* mutant, or on still unknown genes (see Fig. 4b).

On the other hand, we think that the excess of phosphorylated RNA pol II molecules without transcriptional activity associated to ORF-containing genes over the active ones could be a general feature of yeast genes. In a previous paper we showed that some functional categories, such as RP genes had an excess of backtracked RNA pol II molecules [35]. This result was deduced from the comparison between RPCC with Ser5-phosphorylated-specific antibody, that detects all RNA pol II molecules having this post-translational modification onto their CTD tail, and GRO, that detects only transcriptionally active ones. However, in that study we were only able to determine that RP genes were above the general behavior but we cannot quantify the absolute number of RNA pol II molecules that are unable to elongate. This data suggests that, at least, part of the vast excess of phosphorylated molecules (about 11000) that constitute the “transcriptional noise” could be also arrested non-active competent RNA pol II molecules [49]. Another corollary of these calculations is that the RNA pol II density maps that have been published using variants of chip-on-chip technology [42], [50] represent, probably, a compendium of active and non-active (arrested) molecules more than a transcribing map of the yeast genome and that it is necessary to couple those data with measurements of the polymerases activity along the genes [51] to understand the dynamics of the transcription process.

### Conclusion

Our data show that most yeast genes are not being productively transcribed in a snapshot of the cell. A large proportion of the transcription is concentrated in a few genes, in fact, 25% of the total transcription is due only to 5% of the genes with the highest TR. This coincides with previous calculations that indicated that many mRNAs present in a cell are inherited from the mother [52]. Assuming that the 4670 genes, from which we obtained TR data, are representative of the 5796 non dubious ORF-containing genes, it is possible to calculate approximately 1000 productive transcription events (leading to the production of new mature mRNAs) occurring each minute in a yeast cell. This means that 113500 events take place in a typical cell cycle. Given that the current data for mRNA molecules per yeast cell is thought to be 26000 [3], the turnover of the whole set of mRNAs would be more than four times per cell cycle.

## Materials and Methods

### Respective contributions of mRNA degradation and dilution to the disappearance of mRNA during cell growth

Taking into account the mRNA amount for each gene and the cellular generation time, it is possible to compute the mRNA decrease inside the cell due just to the cell doubling, and thus the TR devoted to compensate this effect. In our hands the BY4741 strain has a doubling time of 113 min in exponential phase in YPD at 28°C. We computed the decrease in the mRNA concentration due to the dilution for each gene as RA*(ln2/doubling time). For this we computed a new mRNA amount dataset derived from the RNA-Seq data [4], which had been normalized to real units (mRNAs/cell) using the 2000 most abundant mRNAs from the GATC-PCR data [3].

### Generation of a nascent transcription rate dataset

An averaged GRO dataset was generated using the ArrayStat software and 8 different GRO experiments of exponentially growing cells done in triplicate (for a total of 24 independent biological samples) with a minimum Pearson correlation among them of 0.7. Only those genes with at least 5 valid measures throughout the 8 different experiments were accepted. The GRO analyses were done as previously described [10]. Briefly, the signals from ^33^P-UTP labeled run-on samples were normalized using the signals from the same filters hybridized with ^33^P-dCTP random primer labeled genomic DNA samples.

In order to remove the gene length dependent bias (see Text S1), two different lowess smoothing analyses were carried out for those genes with lengths above or below 3 kb. Specifically, the z-score values on the logarithmic scale were computed for both GRO and RPCC data. The datasets were changed to log_2_ scale and to each individual value the population mean was subtracted. Then, this difference was divided by the standard deviation. Afterwards, the z-score values on the logarithmic scale were represented against the ORF length (Fig. S1). Two independent lowess smoothers were performed for the ORF above or below 3 kb using a smoother span of 20% of the data. The GRO data were normalized subtracting to each value the difference between the GRO and RPCC lowess smoothers in order to correct any probe length bias. Finally, the GRO standardization was reversed (multiplying each value by the original standard deviation and adding the population mean).

To transform the TR in arbitrary units into real ones (molecules/min), a general steady-state for the gene expression was assumed [21]. First, a indirect TR dataset was computed using genomic mRNA half-life data [17] (in minutes) and the newly computed mRNA amount dataset described above. However, this indirect TR dataset was computed using non-growing cells [17]. Thus, in order to incorporate the dilution effect (decrease in the RA due to the cellular dilution) we added to each gene the TR needed to compensate the dilution ([RA*(ln2/generation time)], as computed before). Finally, the nascent TR data, in arbitrary units, previously obtained in this study were normalized to real units (molecules/min) using the 2000 genes with the highest indirect dilution-corrected TR.

### Correlations of the TR datasets with some chIP datasets

To test the improvement of the new nascent TR dataset and it relationship with modifications associated to active transcription we compared the new data with some alternative transcription datasets. These datasets include a newly computed indirect TR dataset using genomic mRNA half-life data [17] and new estimations of RA [3], [4] (see above), the original indirect TR computed by Holstege *et al.* [9] and the new estimation of mRNA amount. We compared those datasets with data from [25], and computed the Spearman correlation.

### Comparison between nascent TR and indirect TR datasets

In order to compare the nascent TR dataset with the indirect one, we used a Log_2_(TR/indTR) dataset in which the general bias due to the different transcription datasets amplitudes was corrected. Specifically, we plot Log_2_(TR/indTR) against Log_2_(TR + indTR) and corrected the bias related with the TR using a lowess smoothing with a smoother span of 20% of the data.

### Accession numbers

All the experiments were done in triplicate. Genomic data are stored in the Valencia Yeast (VYdBase; http://vydbase.uv.es/) and the GEO databases. GEO series accession numbers are GSE14060 for RPCC hybridizations, and GSE11521 and GSE13096 for GRO data.

## Supporting Information

Text S1 **Supplementary analysis and references.** (DOC)

Table S1 **Whole dataset of the nascent TR obtained in this work and other transcription parameters.** It is presented the nascent (TR) and the indirect transcription rates (TR indirect) computed from [17]. Both data are shown before and after the correction used to compensate the dilution effect. (XLS)

Table S2 **Nascent TR datasets from to other physiological conditions.** We used previously published GRO datasets to expand the change from cells growing in glucose to galactose [10] oxidative stress [36] and osmotic stress [37]. (XLS)

Table S3 **Percentage of TR devoted to compensate the dilution effect due to the cell growth.** The TR necessary to compensate the dilution is computed using the RA and a generation time of 113 minutes. As the TR necessary to compensate the dilution is computed independently to the nascent TR some values show percentage greater than 100% or are negative. In those cases we have arbitrary substituted the values to either 100% or 0%, meaning that the TR devoted to compensate the dilution is much larger (100%) or negligible (0%) in respect to the TR devoted to compensate the degradation. (XLS)

Figure S1 **Transcription data biases due to the ORF and probe length.** A) Comparison between RPCC (red), GRO (blue, data from [10]) and random-priming cDNA data (green, data from [10]). B) Confirmation of the 3′ labeling bias using an oligo-dT cDNA labeling (from ref. [S2], purple) and random-priming cDNA data as a control (green). All the curves represent the smoothness of the data using the averages values for a sliding window of 100 genes. The 3 kb ORF length (vertical red line) marks a change in the probe length design for the arrays from the complete ORF for genes shorter than 3 kb to the 3′ last 1 kb in longer genes [S3]. C) Lowess correction (yellow) of the probe length dependent bias for the GRO data (blue) using RPCC data (red). All the values are presented as z-score standardized arbitrary units. (PDF)

Figure S2 **Comparison of the direct and indirect TR datasets with chromatin modifications related to active promoters.** Comparison of the new direct TR dataset (this study) and indirect TR datasets computed using the mRNA amount and the mRNA stability [9], [17] as in Fig. 1 with chromatin modification associated with active promoters as H3K9 acetylation (A) or H3K14 acetylation (B) and H3K4 trimethylation [25]. To avoid any bias depending on the wideness of the datasets Spearman rank correlation is used. Note that no clear improvement in the correlation indexes exists when comparing nascent and indirect datasets. For simplicity, we have used the average values for the probes covering the coding region as indicator of the chromatin modification or protein binding, or when using RA dataset. (PDF)

Figure S3 **Comparison between nascent and indirect transcription rates.** Comparison of the new direct TR dataset (this study) and indirect TR datasets computed using the mRNA amount and the mRNA stability [9], [17]. Note than the correlation is higher when comparing the two indirect TR datasets (C) than when comparing direct and indirect datasets (A, B). Spearman rank correlation is showed. (PDF)

Figure S4 **Differences between nascent and indirect transcription rates according to the overlap with non-coding transcripts.** The box plot shows the relationship Log_2_(TR/indTR) after lowess correction (see Methods section) for ORFs overlapping with CUTs (red), SUTs (blue) or without any overlapping transcript (bisque) according to [29]. The box plots show the median and quartiles of the data. The whiskers show the maximum and minimum of the data set (excluding the outliers which lie beyond 1.5 times the inter quartile range). The significance values represent the p value for the t test of difference of the mean. (PDF)

Figure S5 **Differences between nascent and indirect transcription rates according to RNA pol II density across the genes.** The box plot shows the relationship Log_2_(TR/indTR) after lowess correction (see Methods section) for genes classified according to their distribution of RNA pol II along the gene. It is shown cluster 1 (red, genes with accumulation of non-elongating polymerases in the promoter region), cluster 2 (blue, genes with enriched levels of RNA pol II molecules on their 5′ end transcribed region) and cluster 3 (bisque, genes with an even or enriched to the 3′ distribution of polymerases) in reference to the rest of the analyzed genes (white boxes). See Venters and Pugh [24] for cluster details. The box plots show the median and quartiles of the data. The whiskers show the maximum and minimum of the data set (excluding the outliers which lie beyond 1.5 times the inter quartile range). The significance values represent the p value for the t test of difference of the mean. (PDF)

Figure S6 **Functional analysis of the TR using the FatiScan tool.** (Babelomics software, [S3]). GOs in red represent the groups associated with high TRs (either overrepresented groups in genes with high TRs (left) or underrepresented groups in genes with low TRs (right)). GOs in blue represent the groups associated with low TRs (either overrepresented groups in genes with low TRs (right) or underrepresented groups in genes with high TRs (left)). Only GOs with a statistically significant difference from the population is shown (multitest corrected p-value <0.001). The three parts represent independent searches for GOs in “biological process”, “molecular function” and “cellular component”, in that order. (PDF)

Figure S7 **Functional analysis of the percentage of TR devoted to compensate the dilution.** (Babelomics software, [S3]). GOs in red represent the groups associated with genes that devote a high percentage of its TR to compensate the dilution due to the cellular growth, whereas in blue are represented those genes in which the contribution of the dilution to the mRNA disappearance is smaller. Only GOs with a statistically significant difference from the population are shown (multitest corrected p-value <0.001). The three parts represent independent searches for GOs in “biological process”, “molecular function” and “cellular component”, in that order. (PDF)
