# Correction: A Complete Set of Nascent Transcription Rates for Yeast Genes

**DOI:** 10.1371/journal.pone.0115560

**Published:** 2014-12-05

**Authors:** The PLOS ONE Staff

There is an error in Supporting Information Table S3. The headings in columns G and H are interchanged. Please view the corrected table below.

## Supporting Information

Table S3 **Percentage of TR devoted to compensate the dilution effect due to the cell growth.** The TR necessary to compensate the dilution is computed using the RA and a generation time of 113 minutes. As the TR necessary to compensate the dilution is computed independently to the nascent TR some values show percentage greater than 100% or are negative. In those cases we have arbitrary substituted the values to either 100% or 0%, meaning that the TR devoted to compensate the dilution is much larger (100%) or negligible (0%) in respect to the TR devoted to compensate the degradation
(XLS)
