# Supplementary material for: A Complete Set of Nascent Transcription Rates for Yeast Genes
Source: PLoS One. 2010 Nov 16;5(11):e15442. doi: 10.1371/journal.pone.0015442 (PMC2982843; doi:10.1371/journal.pone.0015442)
Supplement: Text S1 — Supplementary analysis and references. (DOC) [file pone.0015442.s001.doc]

**Supplementary analysis**

**Detection and correction of the specific probe length data in GRO**

When plotting the GRO and RPCC data against the gene length a negative correlation can be detected between the gene length and the TR. Although the fact that shorter genes tend to be more expressed has already been described [3, 10, 19] the slope of this bias is higher in the GRO dataset (blue) than in the RPCC (red) or a mRNA labeling using random priming (green) (supplemental Fig S1). Moreover, the GRO curve shows a change in tendency for those genes whose ORF was longer than 3 kb. The average line increases to levels higher than 0 with genes reaching levels of about 1 kb in length. The macroarrays used for both RPCC and GRO present for those genes longer than 3 kb a probe that covers only the 1 kb downstream of the ORF, whereas the rest of the genes (<3 kb) are represented by the whole of the ORF [S1]*.* As this technical effect is only observed in the GRO data, this allows to determine that the differences in the slope against the length of the gene is in fact a technical bias specific of the GRO technique

As the GRO method labeled the elongating mRNA by extending it 200-300 nt downstream [13], it is expected to bias the label to the 3’ part of the genes. This would lead to an increased average label in the genes as their length decreases and the relative influence of their 3’ part increases. It also predicts a sudden increase for the genes represented by probes covering only their last 1 kb of the ORF. What this reasoning predicts is that other labeling protocols with a bias to the 3’ end of the mRNA will also present a similar average curve. To confirm this hypothesis we made a similar analysis using the data of mRNA labeled using an oligo-dT primer for the cDNA synthesis. This kind of labeling which mainly labels the 3’ end of the mRNA (purple curve in supplemental Fig S1B), shows a similar profile to that of the GRO curve, while the curve obtained by random priming labeling of cDNA (green curve) is similar to that of the RPCC. With this reasoning, we may conclude that there is a general bias in the GRO that increases the calculated TR inversely with the ORF (i.e., probe) length. Additionally, there is an artifact for ORFs that is longer than 3 kb due to the use of 1 kb probes from their 3’ end. Thus, this gene/prove dependent effect can be corrected by using the RPCC data for lowess normalization of the GRO data.

We analyzed the GRO and RPCC data for other biases related with G+C content, transcription rate or gene length. However we were not able to identify other technical bias that we could unequivocally assign to one of the two methods.

**Supplementary references**

S1 Alberola TM, García-Martínez J, Antúnez O, Viladevall L, Barceló A et al. (2004) A new set of DNA macrochips for the yeast Saccharomyces cerevisiae: features and uses. Int Microbiol 7: 199-206.

S2 Pelechano V, Pérez-Ortín JE (2008) The transcriptional inhibitor thiolutin blocks mRNA degradation in yeast. Yeast 25: 85-92.

S3 Al-Shahrour F, Carbonell J, Mínguez P, Goetz S, Conesa A et al. (2008) Babelomics: advanced functional profiling of transcriptomics, proteomics and genomics experiments. Nucleic Acids Res 36: W341-W346.
