# Supplementary figures and images for: A Complete Set of Nascent Transcription Rates for Yeast Genes
Source: PLoS One. 2010 Nov 16;5(11):e15442. doi: 10.1371/journal.pone.0015442 (PMC2982843; doi:10.1371/journal.pone.0015442)

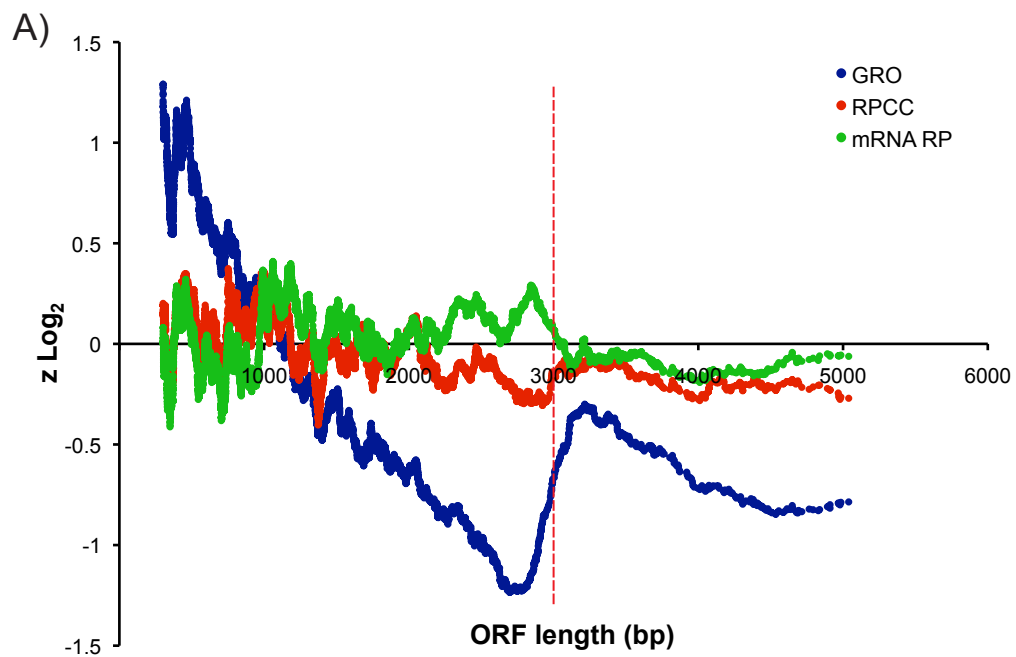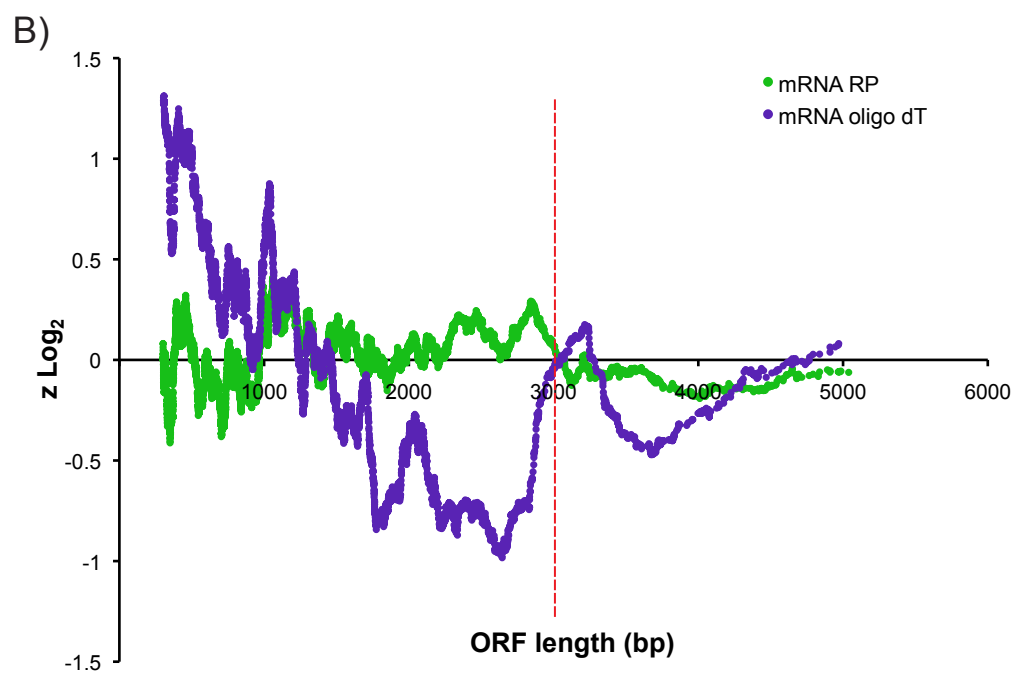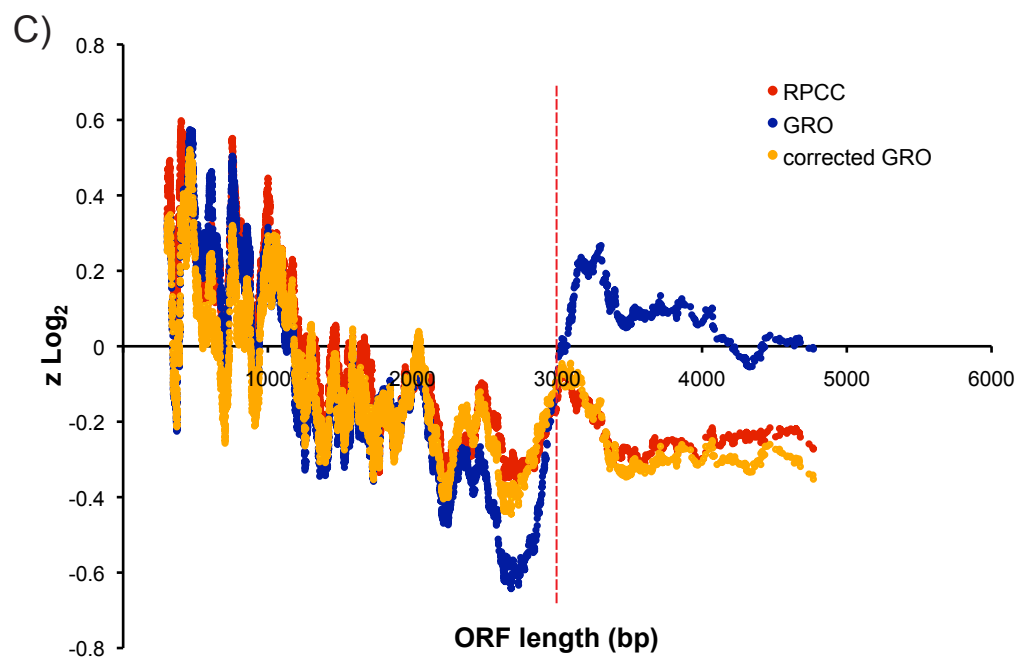

Supplement: Figure S1 — Transcription data biases due to the ORF and probe length. A) Comparison between RPCC (red), GRO (blue, data from [10]) and random-priming cDNA data (green, data from [10]). B) Confirmation of the 3′ labeling bias using an oligo-dT cDNA labeling (from ref. [S2], purple) and random-priming cDNA data as a control (green). All the curves represent the smoothness of the data using the averages values for a sliding window of 100 genes. The 3 kb ORF length (vertical red line) marks a change in the probe length design for the arrays from the complete ORF for genes shorter than 3 kb to the 3′ last 1 kb in longer genes [S3]. C) Lowess correction (yellow) of the probe length dependent bias for the GRO data (blue) using RPCC data (red). All the values are presented as z-score standardized arbitrary units. (PDF) [file pone.0015442.s005.pdf]

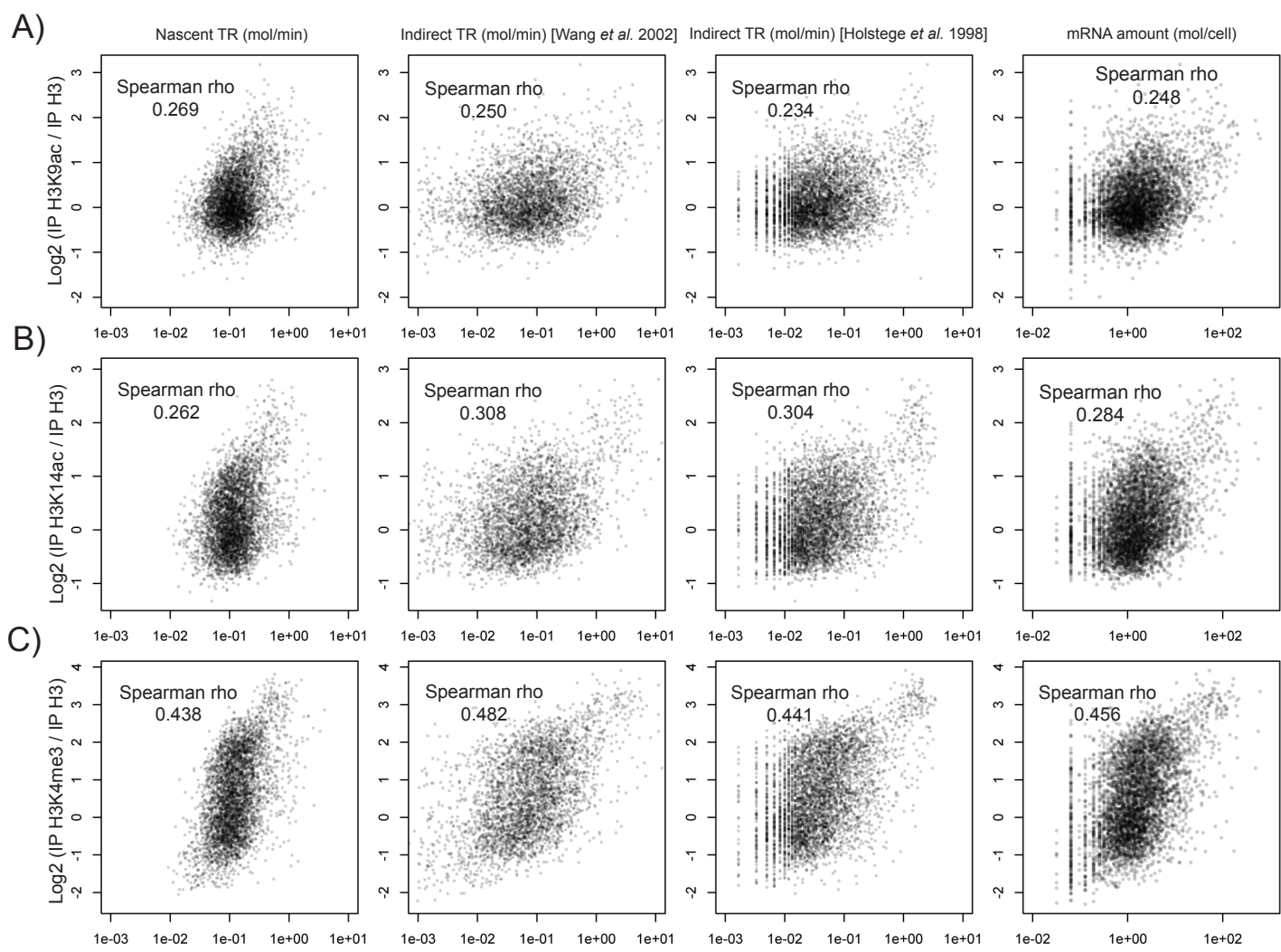

Supplement: Figure S2 — Comparison of the direct and indirect TR datasets with chromatin modifications related to active promoters. Comparison of the new direct TR dataset (this study) and indirect TR datasets computed using the mRNA amount and the mRNA stability [9], [17] as in Fig. 1 with chromatin modification associated with active promoters as H3K9 acetylation (A) or H3K14 acetylation (B) and H3K4 trimethylation [25]. To avoid any bias depending on the wideness of the datasets Spearman rank correlation is used. Note that no clear improvement in the correlation indexes exists when comparing nascent and indirect datasets. For simplicity, we have used the average values for the probes covering the coding region as indicator of the chromatin modification or protein binding, or when using RA dataset. (PDF) [file pone.0015442.s006.pdf]

A)

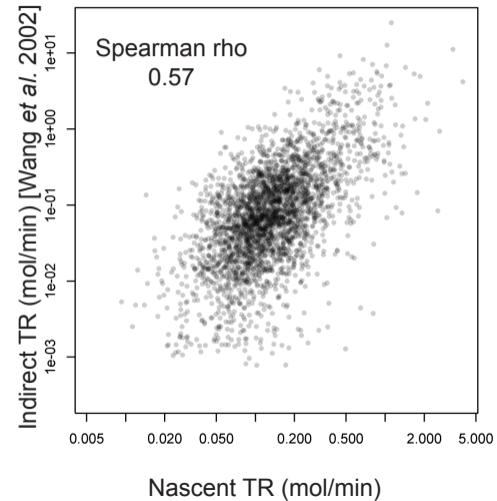

B)

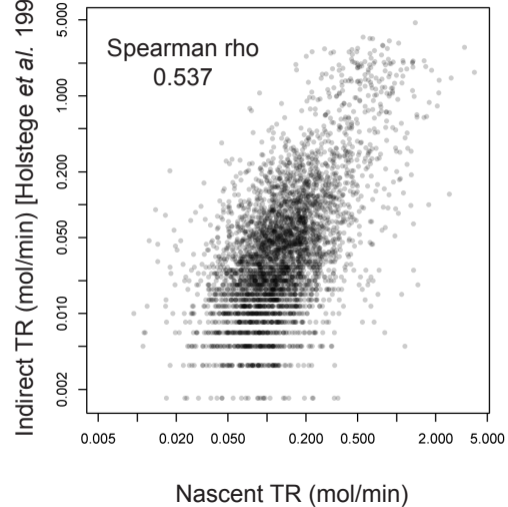

C)

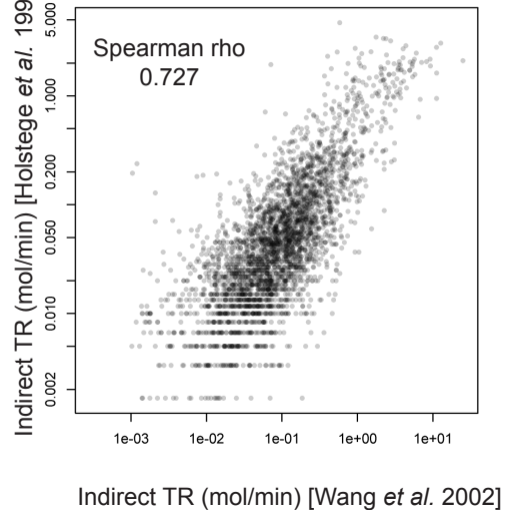

Supplement: Figure S3 — Comparison between nascent and indirect transcription rates. Comparison of the new direct TR dataset (this study) and indirect TR datasets computed using the mRNA amount and the mRNA stability [9], [17]. Note than the correlation is higher when comparing the two indirect TR datasets (C) than when comparing direct and indirect datasets (A, B). Spearman rank correlation is showed. (PDF) [file pone.0015442.s007.pdf]

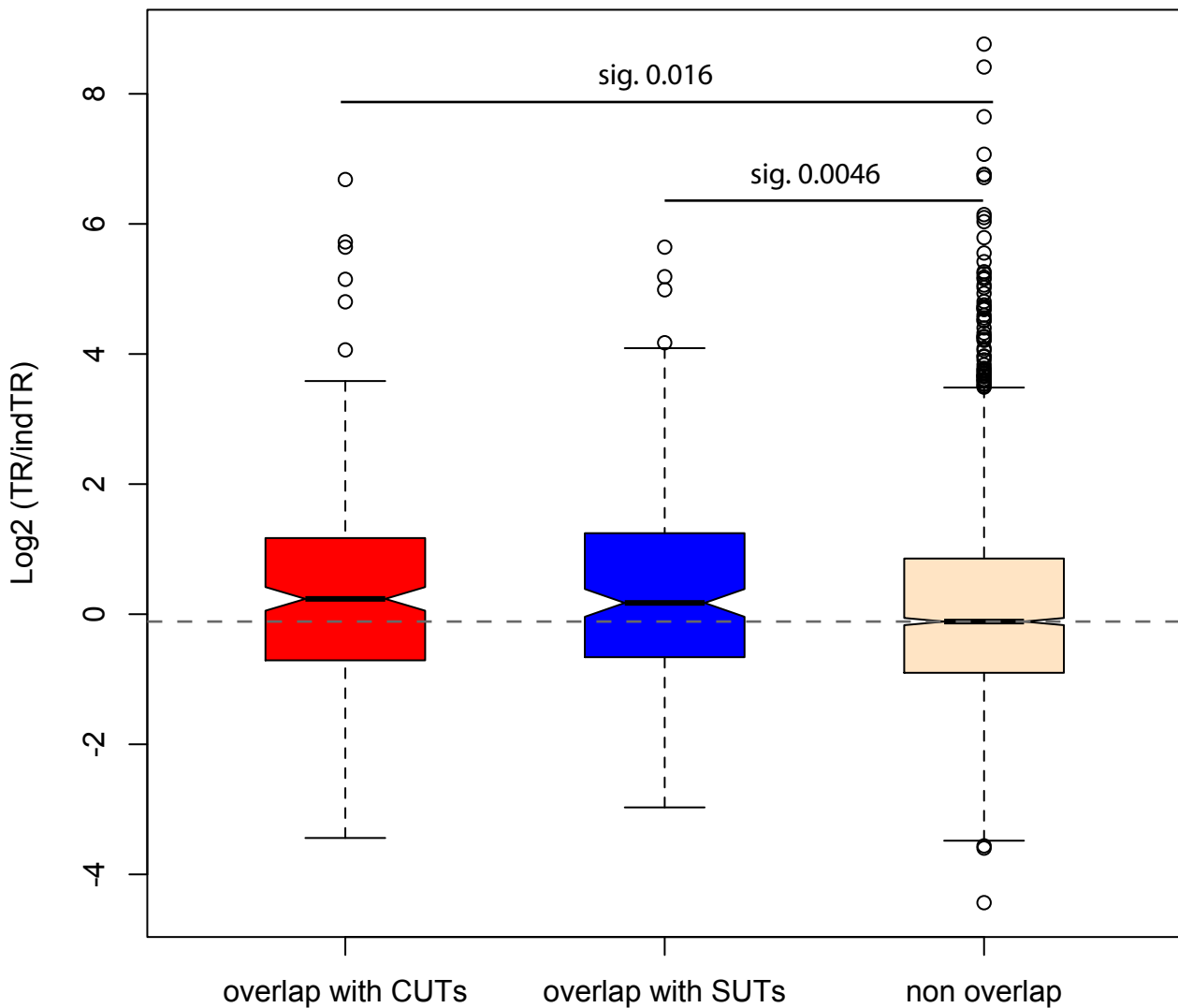

Supplement: Figure S4 — Differences between nascent and indirect transcription rates according to the overlap with non-coding transcripts. The box plot shows the relationship Log2(TR/indTR) after lowess correction (see Methods section) for ORFs overlapping with CUTs (red), SUTs (blue) or without any overlapping transcript (bisque) according to [29]. The box plots show the median and quartiles of the data. The whiskers show the maximum and minimum of the data set (excluding the outliers which lie beyond 1.5 times the inter quartile range). The significance values represent the p value for the t test of difference of the mean. (PDF) [file pone.0015442.s008.pdf]

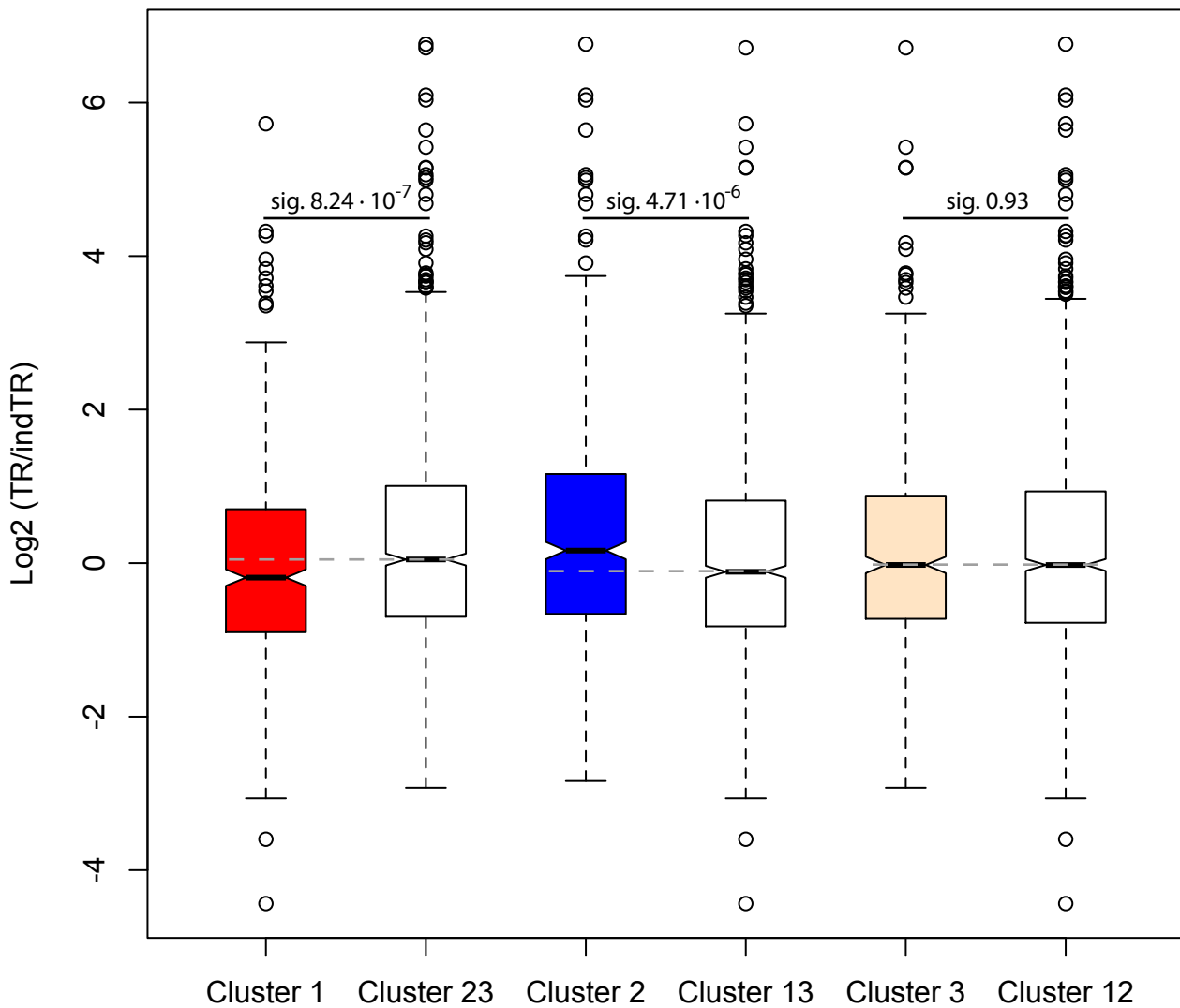

Supplement: Figure S5 — Differences between nascent and indirect transcription rates according to RNA pol II density across the genes. The box plot shows the relationship Log2(TR/indTR) after lowess correction (see Methods section) for genes classified according to their distribution of RNA pol II along the gene. It is shown cluster 1 (red, genes with accumulation of non-elongating polymerases in the promoter region), cluster 2 (blue, genes with enriched levels of RNA pol II molecules on their 5′ end transcribed region) and cluster 3 (bisque, genes with an even or enriched to the 3′ distribution of polymerases) in reference to the rest of the analyzed genes (white boxes). See Venters and Pugh [24] for cluster details. The box plots show the median and quartiles of the data. The whiskers show the maximum and minimum of the data set (excluding the outliers which lie beyond 1.5 times the inter quartile range). The significance values represent the p value for the t test of difference of the mean. (PDF) [file pone.0015442.s009.pdf]
