# Supplementary material for: A Complete Set of Nascent Transcription Rates for Yeast Genes
Source: PLoS One. 2010 Nov 16;5(11):e15442. doi: 10.1371/journal.pone.0015442 (PMC2982843; doi:10.1371/journal.pone.0015442)
Supplement: Figure S6 — Functional analysis of the TR using the FatiScan tool. (Babelomics software, [S3]). GOs in red represent the groups associated with high TRs (either overrepresented groups in genes with high TRs (left) or underrepresented groups in genes with low TRs (right)). GOs in blue represent the groups associated with low TRs (either overrepresented groups in genes with low TRs (right) or underrepresented groups in genes with high TRs (left)). Only GOs with a statistically significant difference from the population is shown (multitest corrected p-value <0.001). The three parts represent independent searches for GOs in “biological process”, “molecular function” and “cellular component”, in that order. (PDF) [file pone.0015442.s010.pdf]

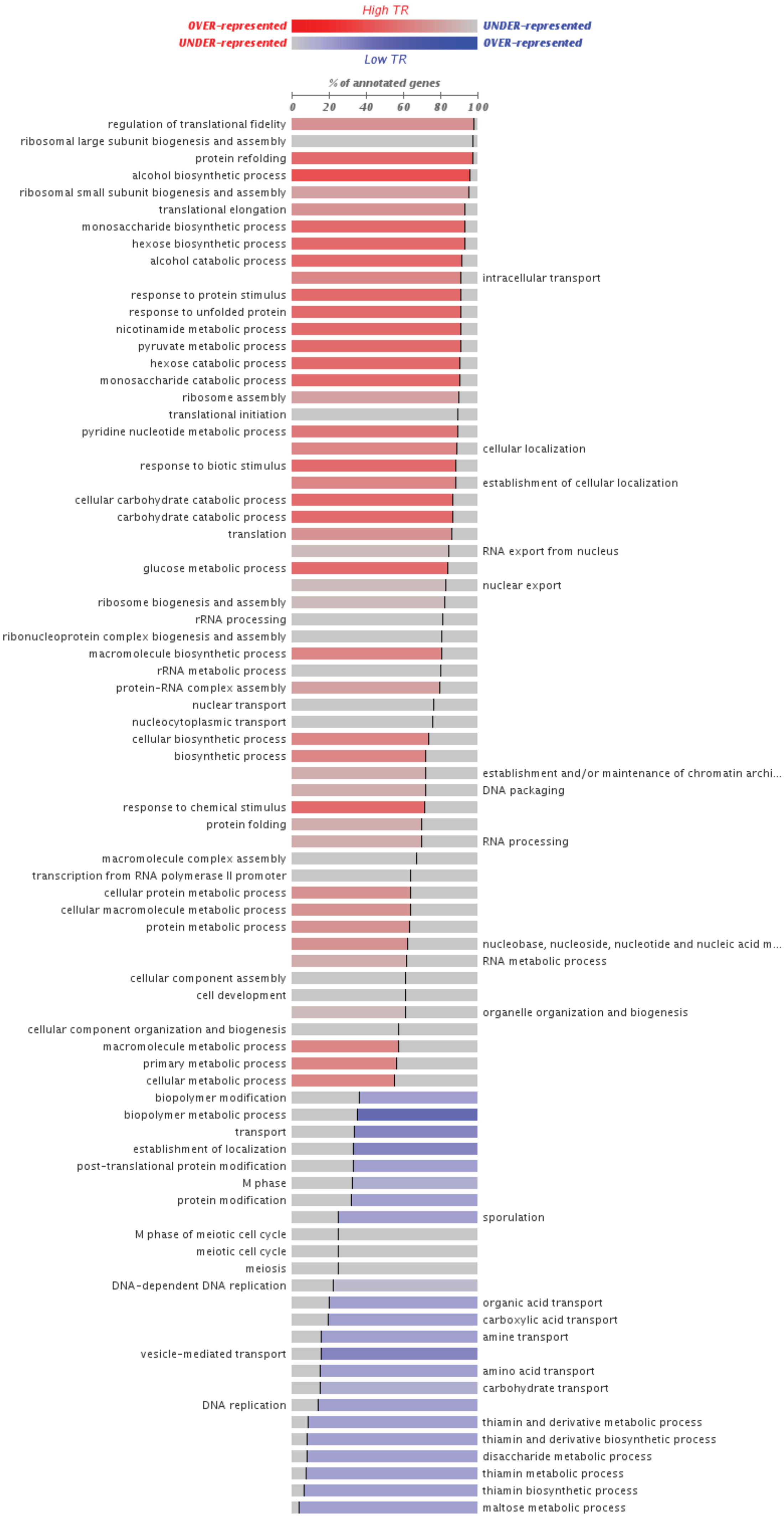

Over/Under term representation in GO biological process between levels 3 to 9

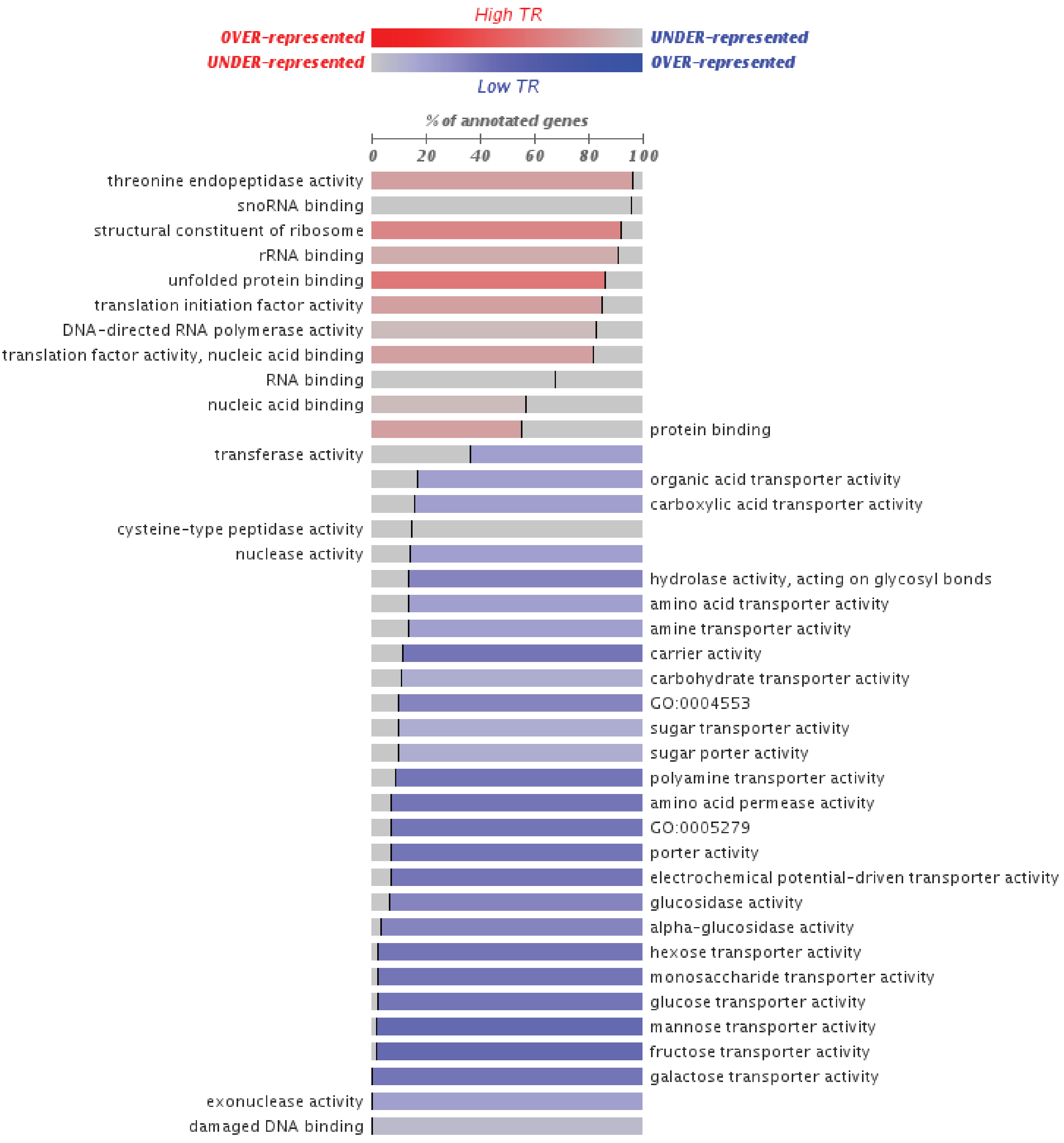

Over/Under term representation in GO molecular function between levels 3 to 9

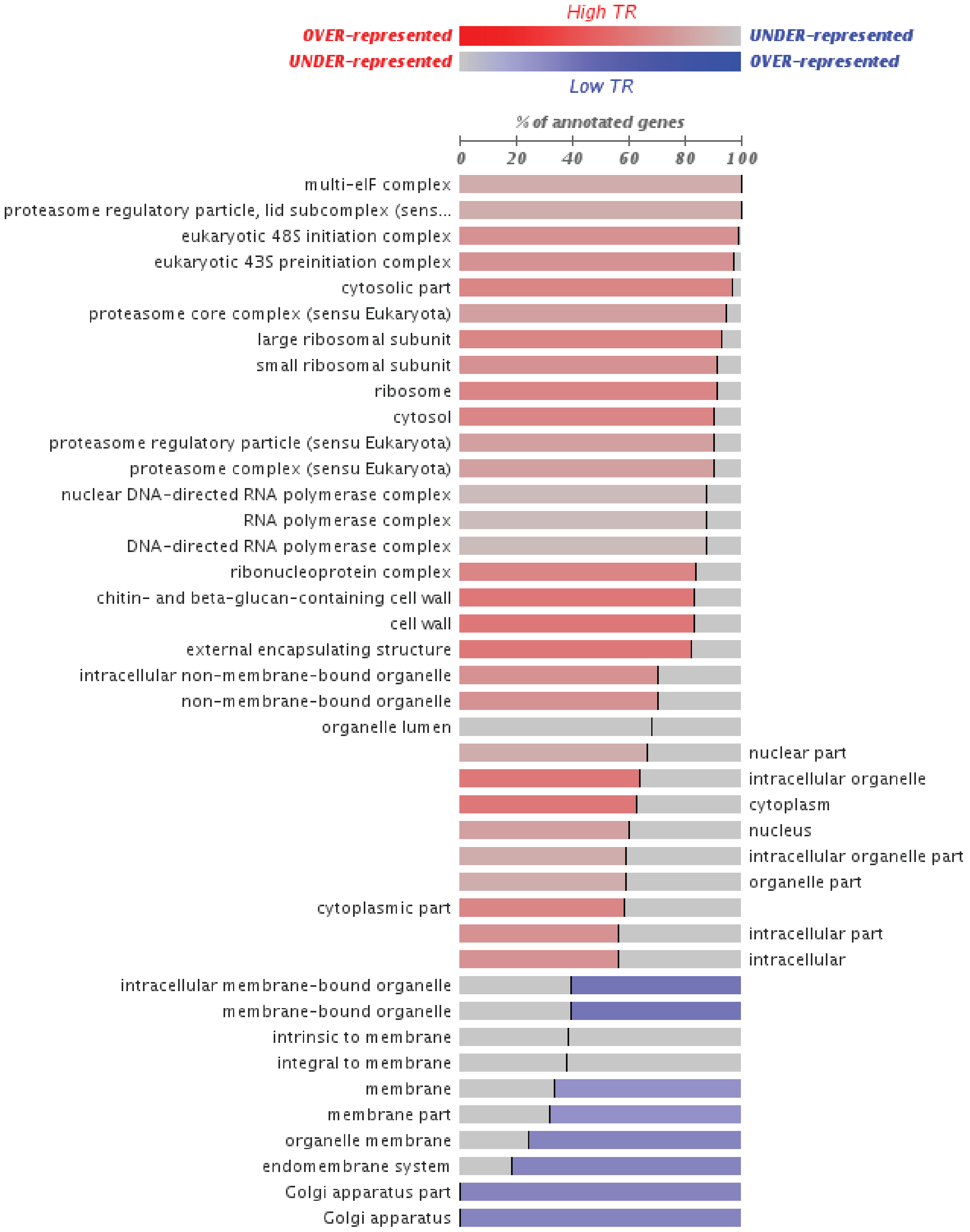

Over/Under term representation in GO cellular component between levels 3 to 9
