# Supplementary material for: A Complete Set of Nascent Transcription Rates for Yeast Genes
Source: PLoS One. 2010 Nov 16;5(11):e15442. doi: 10.1371/journal.pone.0015442 (PMC2982843; doi:10.1371/journal.pone.0015442)
Supplement: Figure S7 — Functional analysis of the percentage of TR devoted to compensate the dilution. (Babelomics software, [S3]). GOs in red represent the groups associated with genes that devote a high percentage of its TR to compensate the dilution due to the cellular growth, whereas in blue are represented those genes in which the contribution of the dilution to the mRNA disappearance is smaller. Only GOs with a statistically significant difference from the population are shown (multitest corrected p-value <0.001). The three parts represent independent searches for GOs in “biological process”, “molecular function” and “cellular component”, in that order. (PDF) [file pone.0015442.s011.pdf]

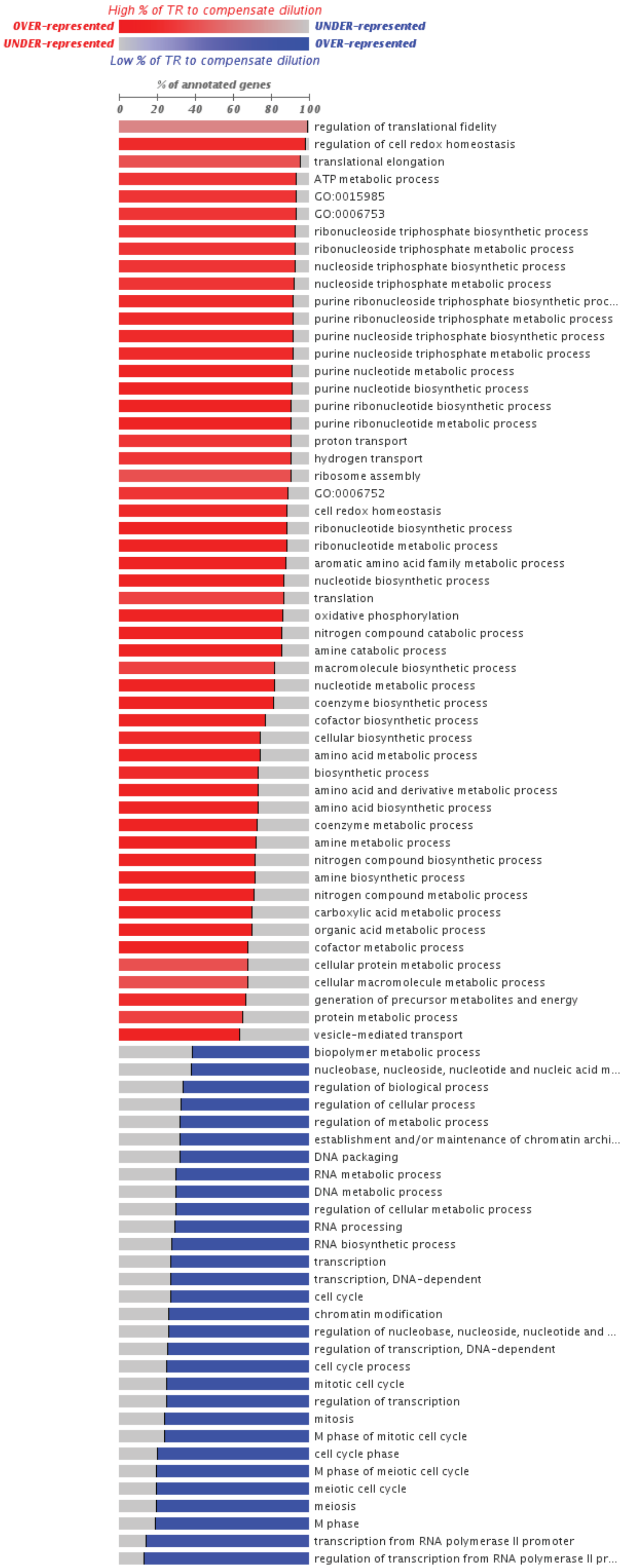

Over/Under term representation in GO biological process between levels 3 to 9

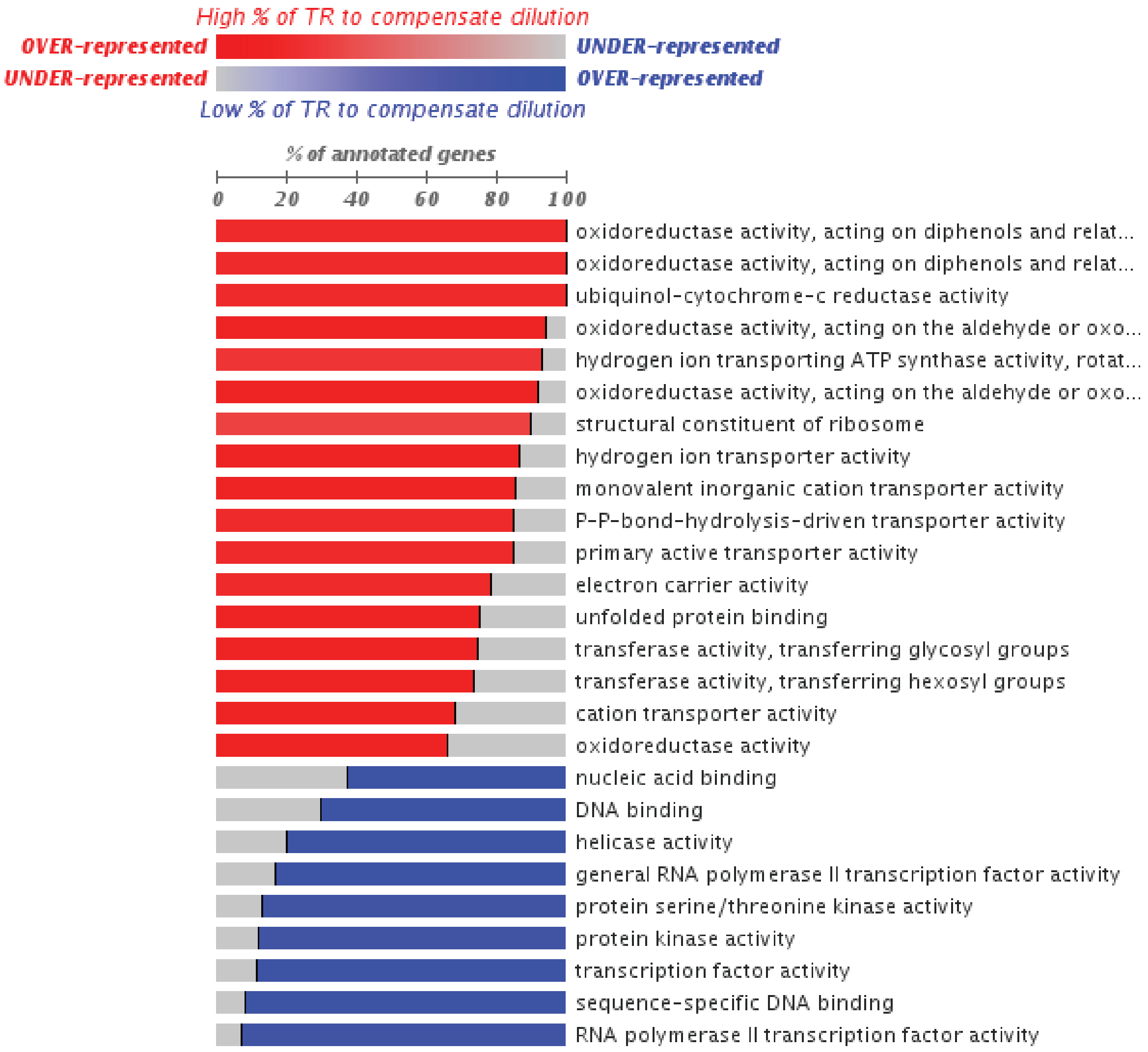

Over/Under term representation in GO molecular function between levels 3 to 9

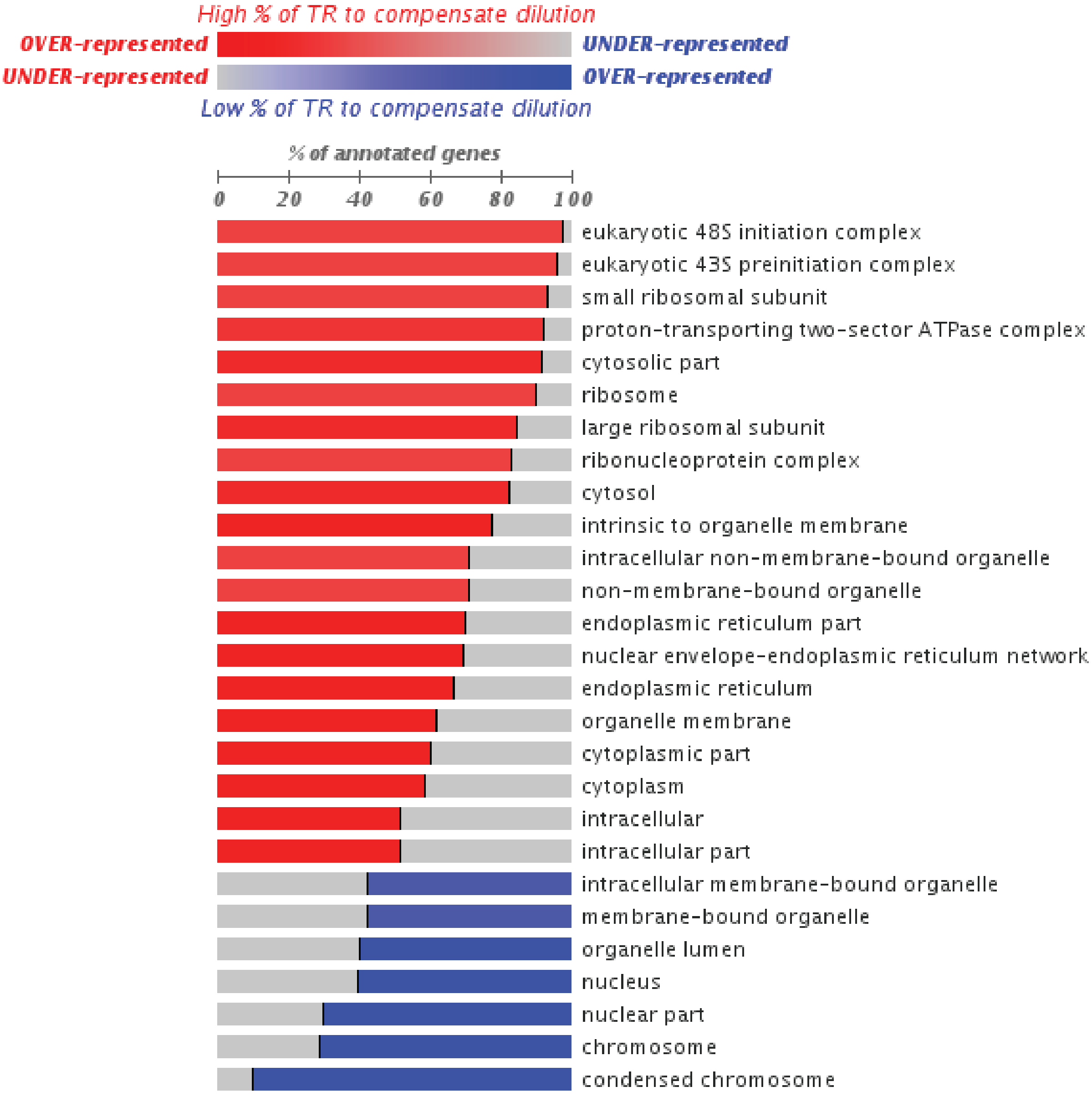

Over/Under term representation in GO cellular component between levels 3 to 9
